# Longitudinal profiling of γδ T cell dynamics after human lung transplantation links donor and recipient subsets to clinical outcomes

**DOI:** 10.3389/fimmu.2026.1811464

**Published:** 2026-04-16

**Authors:** Joshua H. Wang, Tyla Young, Katherine D. Long, Wenyu Jiao, Constanza Bay Muntnich, Adriana Prada Rey, Morcel Khwajazadah, Vineetha Mohan, Kortney Rogers, Arnold Valena, Joseph Costa, Luke Benvenuto, Joshua Sonett, Philippe Lemaitre, Frank D’Ovidio, Selim Arcasoy, Jianing Fu

**Affiliations:** 1 Columbia Center for Translational Immunology, Department of Medicine, Columbia University Irving Medical Center, New York, NY, United States; 2 Columbia College, Columbia University, New York, NY, United States; 3 Department of Nursing, New York-Presbyterian Hospital, New York, NY, United States; 4 Department of Surgery, Columbia University Irving Medical Center, New York, NY, United States; 5 Division of Pulmonary, Allergy, and Critical Care Medicine, Columbia University Irving Medical Center, New York, NY, United States

**Keywords:** chimerism, lung transplantation (LuTX), rejection, repopulation, γδ T cells

## Abstract

**Introduction:**

Long-term survival after lung transplantation lags behind that of other solid organ transplants, underscoring the need to better understand its complex immune responses to prevent complications and improve clinical outcomes. While most post-transplant immune studies on T lymphocytes have focused on αβ T cells, the role of mucosal-tissue-enriched γδ T cells remains largely unexplored in lung transplantation.

**Methods:**

We performed a longitudinal analysis of the presence, chimerism, and phenotype of γδ T cells in 13 lung transplant recipients, tracking their dynamics in peripheral blood and bronchoalveolar lavage (BAL) for up to 3 years after transplantation.

**Results:**

Patients undergoing bilateral transplantation exhibited a significantly higher percentage of γδ T cells among total T cells in BAL compared to single-lung recipients. In circulation, higher peak levels of donor-derived γδ T cells were associated with a trend toward higher circulating donor γδ T cell counts and lower incidence of acute cellular rejection, suggesting a protective systemic role. While within BAL, a more rapid turnover of recipient γδ T cells was associated with significantly delayed onset of infection and improved pulmonary function. Over time, recipient γδ T cells in BAL showed a phenotypic shift from effector (CD28^high^) toward tissue-resident memory (TRM; CD69^+^/CD103^+^/CD49a^+^) phenotypes. As expected, TRM phenotypes were more prevalent in BAL than in blood for both donor and recipient γδ T cells. Interestingly, rapidly infiltrating recipient γδ T cells in BAL were less TRM-like. Furthermore, infection was associated with an enrichment of recipient-derived effector memory γδ T cells in circulation, suggesting their involvement in blood-graft crosstalk.

**Discussion:**

Overall, our longitudinal study demonstrates the distinct local and systemic dynamics of donor and recipient γδ T cells after human lung transplantation and identifies several phenotypes and kinetics linked to clinical outcomes.

## Introduction

In the United States, lung transplant (LuTx) recipients have a median survival of only 5.8 years, with 1- and 5-year survival rates of 88.5% and 59.7%, respectively (1). These outcomes are consistently inferior to those of other major solid organ transplants, such as kidney and heart grafts (2, 3). This disparity underscores the urgent need for research to prevent complications—including rejection, infection, and chronic lung allograft dysfunction—and to improve long-term outcomes.

A critical but often overlooked factor in the post-LuTx immune response is the substantial pool of donor lymphocytes transferred with the lung allograft (4). Similar to the intestine, the lung contains abundant resident immune cells—including T cells, B cells, and antigen-presenting cells (APCs)—that can drive bidirectional alloreactivity after transplantation (5, 6), encompassing both host-versus-graft (HvG) and graft-versus-host (GvH) responses. This contrasts sharply with other solid organ transplants, such as kidney and heart, where donor grafts carry minimal passenger lymphocytes. Our group has developed flow cytometry-based methods to distinguish donor- from recipient-derived lymphocytes (7) and to quantify these bidirectional immune dynamics after human transplantation (8). These approaches have elucidated mechanisms of graft rejection and acceptance, first in human intestinal transplantation (5, 7–14) and more recently for αβ T cells after lung transplantation (6). Despite these advances in mapping donor-recipient lymphocyte dynamics, particularly for αβ T cells (4, 6), the behavior of γδ T cells within this bidirectional post-LuTx immune landscape remains understudied. Here, we apply this same immune-monitoring framework to longitudinally characterize the presence, chimerism, and phenotypes of donor- and recipient-derived γδ T cells in BAL and peripheral blood after lung transplantation.

γδ T cells are a subset of unconventional T cells that possess both innate and adaptive immune characteristics (15). In humans, they are commonly divided into Vγ9Vδ2 and non-Vγ9Vδ2 populations. The latter are heterogeneous in V gene usages; for example, Vδ1 cells are enriched in barrier and mucosal tissues (including the lung) and often adopt tissue-adapted programs, whereas Vγ9Vδ2 cells are typically more abundant in peripheral blood and can mount rapid, innate-like responses during infection (16). The roles of γδ T cells in transplantation outcomes are controversial, and their underlying mechanisms remain unclear (17–19).

In particular, a comprehensive temporal and spatial map of γδ T cells after human LuTx is still lacking (20). Little is known about the distribution and longitudinal dynamics of donor- versus recipient-derived γδ T cell populations in the peripheral blood and the lung allograft—compartments that reflect systemic and local immunity, respectively. Therefore, our study provides a unique longitudinal resource that defines the presence, chimerism, and phenotypic landscape of these cells. By linking the dynamics and phenotypes of donor and recipient γδ T cells to clinical outcomes—including rejection, infection, and pulmonary function—we reveal key pathophysiological correlates for this understudied population in human LuTx. This work paves the way for future mechanistic investigations into these polyfunctional lymphocytes.

## Methods

### Human lung transplant patient recruitment and sample collection

All procedures in this study were approved by the Columbia University Medical Center Institutional Review Board (IRB protocols AAAS6206 and AAAR2681). Thirteen adult lung transplant recipients were prospectively enrolled in a non-interventional study (**Supplementary Table 1**) after providing written informed consent. Transplantation occurred between November 2020 and October 2022. The cohort had a median age of 55 years and was 53% male. Six patients (patients 1, 2, 3, 7, 11, 12) received single lung transplants, and six patients (patients 4, 5, 6, 8, 10, 13) received bilateral lung transplants. Patient 9 underwent combined bilateral lung and liver transplantation. All patients received lifelong immunosuppression per institutional protocol, including a regimen of tacrolimus, prednisone, mycophenolate mofetil, azathioprine, sirolimus, and/or belatacept. Patients 2 and 5 died within six months post-transplantation and had limited specimen collection; they were therefore excluded from cohort-level analyses. Fresh BAL specimens (10–25 mL) were collected during five protocol bronchoscopies at approximately 0.5, 1, 3, 6, and 12 months post-transplant (post-Tx) for routine clinical follow-up. Additional BAL samples were obtained for clinical indications, including suspected rejection or infection. Protocol peripheral blood draws (10–20 mL) were performed 2–3 times more frequently than scheduled bronchoscopies. Spirometry was conducted up to twice per month after transplantation. To assess pulmonary function, we longitudinally monitored the forced expiratory volume in one second (FEV1) as a percentage of the baseline value. Acute cellular rejection (ACR) was diagnosed and graded on transbronchial biopsies according to International Society for Heart and Lung Transplantation (ISHLT) criteria. Grading was based on the presence and severity of perivascular and interstitial mononuclear infiltrates (Grade A0–A4) and airway inflammation (Grade B0–B2R). Peripheral blood from healthy donors used as flow cytometry controls was obtained through the New York Blood Center.

### Lymphocyte isolation and staining

Peripheral blood mononuclear cells (PBMCs) were isolated from patient blood using density gradient centrifugation with Histopaque-1077. Residual red blood cells were lysed with ACK lysis buffer. Cells from BAL samples were pelleted by centrifugation and resuspended in phosphate-buffered saline (PBS). Isolated cells were stained for multicolor flow cytometry as previously described (7). The staining panel included a set of donor and/or recipient HLA-allotype specific markers (**Supplementary Table 2**; e.g., HLA-A3 APC [R: recipient] and HLA-A2/A28 biotin-Streptavidin(SA)-BUV737 [D: donor]) and a comprehensive panel of immune cell markers (**Supplementary Table 3**): CD45 PE-CF594, CD14 FITC or CD14 APC-Cy7, CD19 BUV496, CD56 BV605, CD3 PerCP-Cy5.5, γδ TCR PE-Cy7, CD4 AF700, CD8 APC-Cy7 or CD8 biotin–SA-BUV737, CD45RA BV510, CCR7 PE, CD69 BV650, CD103 BV711, CD49a BUV395, NKG2D APC (in the absence of HLA-A3 APC), CD28 Pac Blue, and HLA-ABC BV786. Viability was assessed using DAPI. Stained samples were acquired on a Cytek Aurora spectral flow cytometer. Full-spectrum fluorescence data were analyzed to quantify cell size, granularity, and marker expression intensity.

### Data analysis and statistical tests

Immune cell populations, including αβ and γδ T cells, were identified and analyzed using FlowJo software (v10). Donor and recipient chimerism and phenotypes were examined in PBMC and BAL samples. Phenotypic definitions were based on the following marker combinations: 1) Naive/memory subsets (CD45RA vs. CCR7): naïve (CD45RA+CCR7+), central memory (TCM; CD45RA−CCR7+), effector memory (TEM; CD45RA−CCR7−), and terminally differentiated effector memory (TEMRA; CD45RA+CCR7−); 2) TRM subsets: CD69+CD103+/- or CD69+CD49a+/-; 3) Effector T cells (Teff): CD28+NKG2D+/−. For reliable chimerism and phenotypic analysis, data points were excluded if the absolute count of donor- or recipient-derived γδ T cells was below 50 in PBMC samples or below 20 in BAL samples.

Quantification and statistical analysis were performed using GraphPad Prism. Longitudinal data for γδ T cell presence and chimerism were summarized as the area under the curve (AUC), normalized by the total observation period (from first to last post-operative day, POD). Median values were also calculated as an alternative measure of central tendency when applicable. Normally distributed data were compared using an unpaired two-tailed t-test (two groups) or one-way analysis of variance (ANOVA) with Tukey’s *post hoc* test (≥ three groups). Non-normally distributed data were compared using the Mann-Whitney U test (two groups) or Kruskal-Wallis test with Dunn’s *post hoc* test (≥ three groups). Time-to-event outcomes (e.g., first occurrence of ACR or infection) were analyzed using Kaplan-Meier curves, with time measured in POD from transplantation. Participants without an event were censored at the last follow-up date or at a pre-specified cutoff of POD800. Survival curves were compared using the log-rank (Mantel-Cox) test. The normalized AUC for %FEV1/baseline FEV1 was calculated and analyzed in relation to the median recipient γδ T cell chimerism in BAL using the regression ANOVA F-test. For all analyses, p<0.05 indicates statistical significance.

## Results

### Post-LuTx quantification of γδ T cells among total T cells in BAL reveals higher frequencies in bilateral transplant recipients

**Figure 1:**
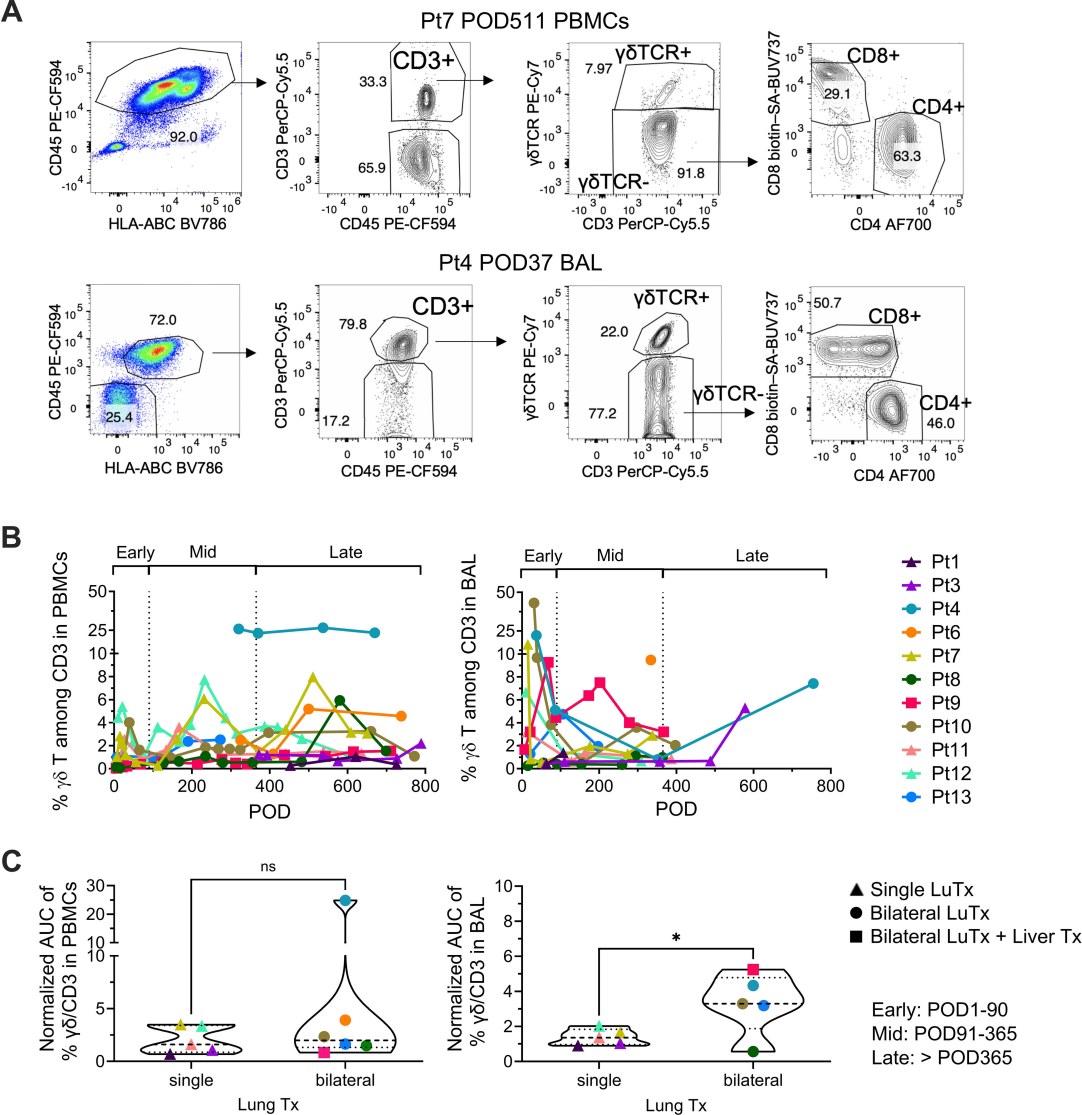
γδ T cell frequencies in PBMCs and BAL following LuTx. **(A)** Representative gating strategy for PBMCs (Pt7, post-operative day [POD]511) and BAL (Pt4, POD37). Sequential gating identified CD45+ HLA-ABC+ leukocytes, CD3+ T cells, and γδTCR+ cells. CD4 and CD8 subsets are shown within the CD3+γδTCR- conventional T cell population. Numbers indicate the percentage of the parent gate. **(B)** Proportion of γδ T cells among total CD3+ T cells in PBMCs (left) and BAL (right), stratified into early (POD1–90), mid (POD91–365), and late (>POD365) post-Tx intervals. **(C)** Comparison of the proportion of γδ T cells among total T cells in PBMCs (left) and BAL (right) between single and bilateral LuTx recipients. Unpaired two-tailed t test was performed for panel **(C)** *p<0.05.

To investigate the presence and dynamics of total γδ T cells after LuTx, we performed longitudinal flow cytometric analysis of PBMC and BAL samples (**Figure 1A**). γδ T cells were consistently detectable in both compartments post-Tx (**Figure 1B**). In PBMCs, they were present at a stable but low mean frequency of 3.2% of total T cells. In BAL, their frequency was slightly higher, with a mean of 4.2% of total T cells, consistent with the known tissue tropism of this population (15). We quantified the longitudinal presence of γδ T cells using normalized AUC analysis (**Figure 1C**). This revealed that recipients of single LuTx had a significantly lower percentage of γδ T cells among total T cells within BAL compared to bilateral LuTx recipients (p=0.0445). No significant difference was observed in the PBMCs. Notably, clinical outcomes, including survival, ACR episodes, *de novo* donor-specific antibody (DSA) development, and infections, did not differ between the single and bilateral transplant groups (**Supplementary Figure 1**). As a baseline reference, γδ T cells were present at variable levels in multiple donor and recipient tissues pre-Tx, including lung, BAL, lung associated lymph nodes (LLN), and PBMCs (**Supplementary Figure 2**). No significant difference was observed between donor and recipient groups across compartments.

### Higher donor γδ T cell chimerism in blood is associated with higher circulating donor γδ T cell counts and reduced ACR

**Figure 2:**
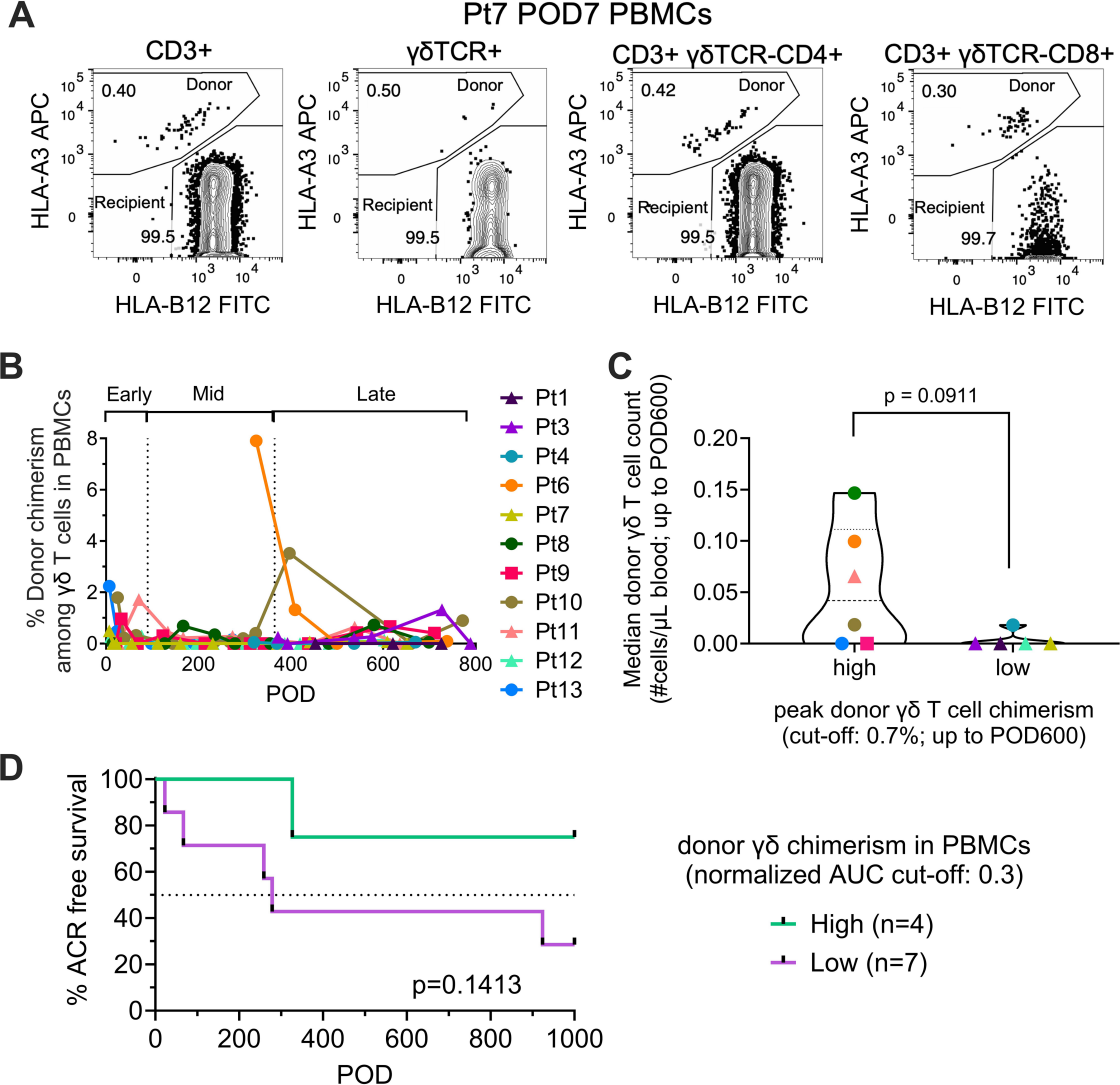
Donor-derived γδ T cell chimerism in circulating PBMCs after LuTx. **(A)** Representative HLA allotype-based gating to quantify donor chimerism within PBMC T cell subsets (Pt7, POD7). Donor- and recipient-derived cells were distinguished by mismatched HLA class I allotypes, with donor cells defined as HLA-A3+HLA-B12- and recipient cells as HLA-A3-HLA-B12+ within the CD3+, γδTCR+, CD3+γδTCR-CD4+, and CD3+γδTCR-CD8+ populations. **(B)** Donor chimerism (%) among circulating γδ T cells in PBMCs, stratified into early (POD1–90), mid (POD91–365), and late (>POD365) post-Tx intervals. **(C)** Median circulating donor γδ T cell counts (cells/µL) up to POD600 in patients with high vs. low peak donor γδ T cell chimerism (cut-off, 0.7%). **(D)** Kaplan-Meier analysis of ACR-free survival, stratified by the normalized AUC of donor γδ T cell chimerism in PBMCs (high vs. low; cut-off, 0.3%). Unpaired two-tailed t test was performed for panel **(C)** Kaplan–Meier analysis with log-rank (Mantel–Cox) test was performed for **(D)**.

Flow cytometric analysis revealed low donor chimerism among peripheral blood γδ T cells, with a mean frequency of 0.35% (**Figures 2A, B**). Patients were stratified into high- and low- chimerism groups based on a peak donor γδ T cell blood chimerism cutoff of 0.7%, which represents the highest detectable chimerism level. The high-chimerism group showed a trend toward higher median donor γδ T cell counts (**Figure 2C**, p=0.0911), suggesting improved engraftment. To account for donor γδ T cell persistence over time rather than just peak levels, patients were further stratified based on a normalized AUC cutoff of 0.3. Kaplan-Meier analysis indicated a trend toward a lower incidence of ACR in the high-chimerism group compared to the low-chimerism group (**Figure 2D**, p=0.1413). The estimated time to 50% ACR-free survival was >1000 days in the high-chimerism group versus <300 days in the low-chimerism group. Stratification thresholds here and in subsequent analyses were empirically defined to balance sample sizes between groups while capturing biological trends. Together, these data indicate that a greater systemic abundance of donor-derived γδ T cells may be associated with a lower incidence of ACR.

### Rapid recipient γδ T cell replacement in BAL is associated with delayed infection and improved pulmonary function

**Figure 3:**
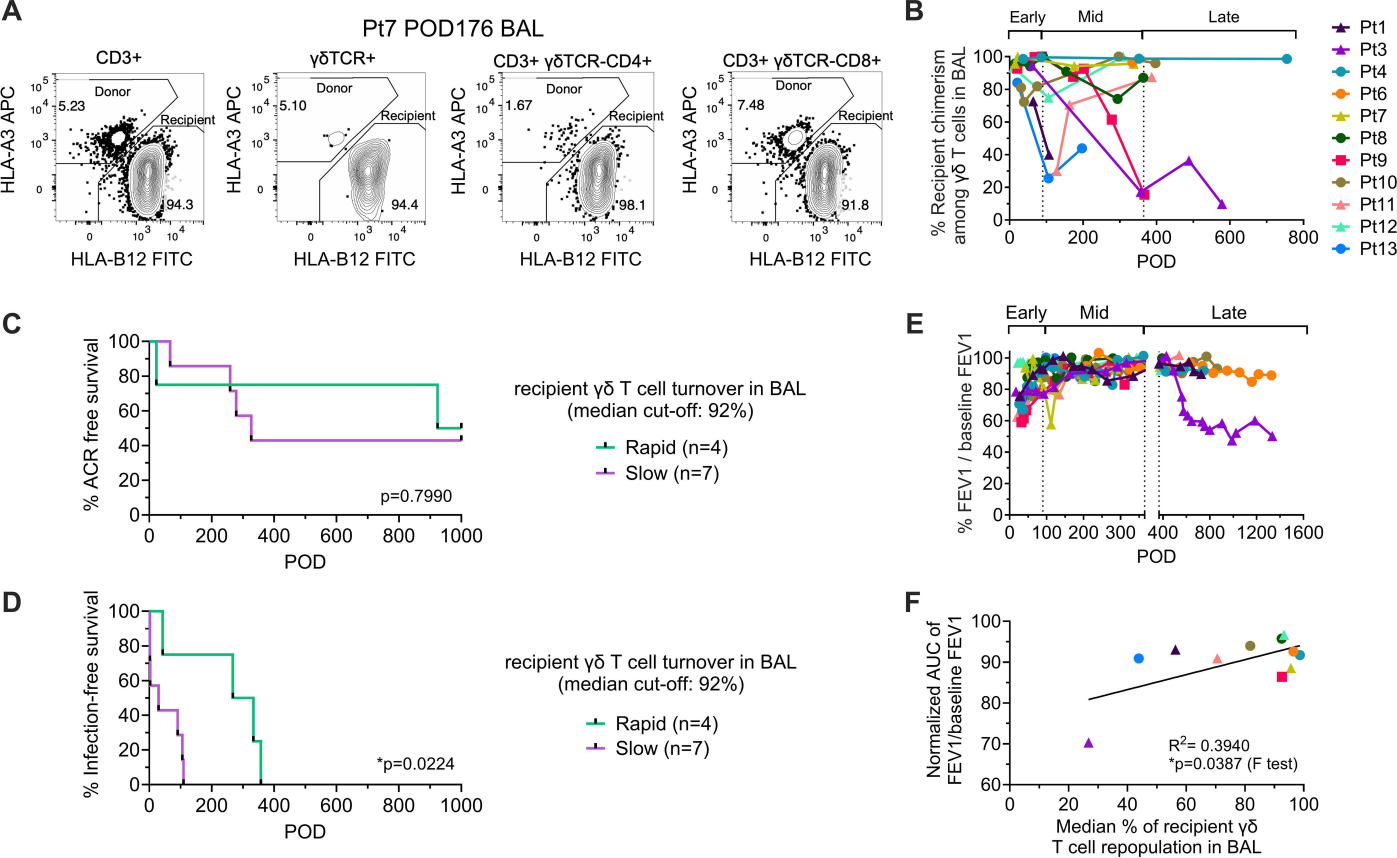
Recipient γδ T cell turnover in BAL and clinical associations after LuTx. **(A)** Representative HLA allotype-based gating to quantify recipient chimerism within BAL T cell subsets (Pt7, POD51). Donor- and recipient-derived cells were distinguished by mismatched HLA class I allotypes, with donor cells defined as HLA-A3+HLA-B12- and recipient cells as HLA-A3-HLA-B12+ within the CD3+, γδTCR+, CD3+γδTCR-CD4+, and CD3+γδTCR-CD8+ populations. **(B)** Recipient chimerism (%) among γδ T cells in BAL, stratified into early (POD1–90), mid (POD91–365), and late (>POD365) post-Tx intervals. Kaplan-Meier analysis of ACR-free survival **(C)** and infection-free survival **(D)**, stratified by recipient γδ T cell turnover in BAL (rapid vs. slow; median cut-off, 92%). **(E)** Longitudinal pulmonary function, expressed as %FEV1/baseline FEV1, over time. **(F)** Association between median recipient γδ T cell repopulation in BAL and the normalized AUC for %FEV1/baseline FEV1. Kaplan–Meier analysis with log-rank (Mantel–Cox) test was performed for **(C, D)** Simple linear regression with ANOVA F test was performed for panel **(F)** *p < 0.05.

Flow cytometric analysis of BAL revealed rapid replacement of donor γδ T cells by recipient γδ T cells, a pattern distinct from the low chimerism observed in peripheral blood (**Figure 3A**). In most patients, recipient γδ T cells dominated the BAL compartment (>80% chimerism) early (<POD90) after transplantation (**Figure 3B**). However, several patients exhibited marked declines in recipient chimerism during the first post-Tx year, showing greater variability than the dynamics of total T cells (6). To account for this fluctuation, recipient chimerism in BAL was quantified using median values. Patients were stratified into rapid- and slow-replacement groups based on a median recipient γδ T cell chimerism cutoff of 92% (**Figures 3C, D**). The incidence of ACR, patient survival, and *de novo* DSA development did not differ between the groups (**Figure 3C**; **Supplementary Figures 3A, B**). In contrast, patients in the rapid-replacement group experienced a significantly delayed onset of infection compared to the slow-replacement group (p=0.0224, **Figure 3D**). To assess pulmonary function, we longitudinally monitored the forced expiratory volume in one second (FEV1) as a percentage of the baseline value (**Figure 3E**). The normalized AUC for %FEV1/baseline FEV1 was calculated and analyzed in relation to the median recipient γδ T cell chimerism in BAL (**Figure 3F**). This analysis revealed that the rapid replacement of recipient γδ T cell in BAL was significantly associated with better preserved pulmonary function (p=0.0387, **Figure 3F**). We did not observe any significant association of recipient γδ T cell turnover dynamic in BAL with donor or recipient age (**Supplementary Figures 3C, D**). Together, these findings suggest that rapid repopulation of the lung allograft by recipient γδ T cells may enhance local mucosal immunity.

### γδ T cells in BAL display a phenotypic shift from effector to tissue-resident memory T cells over time

**Figure 4:**
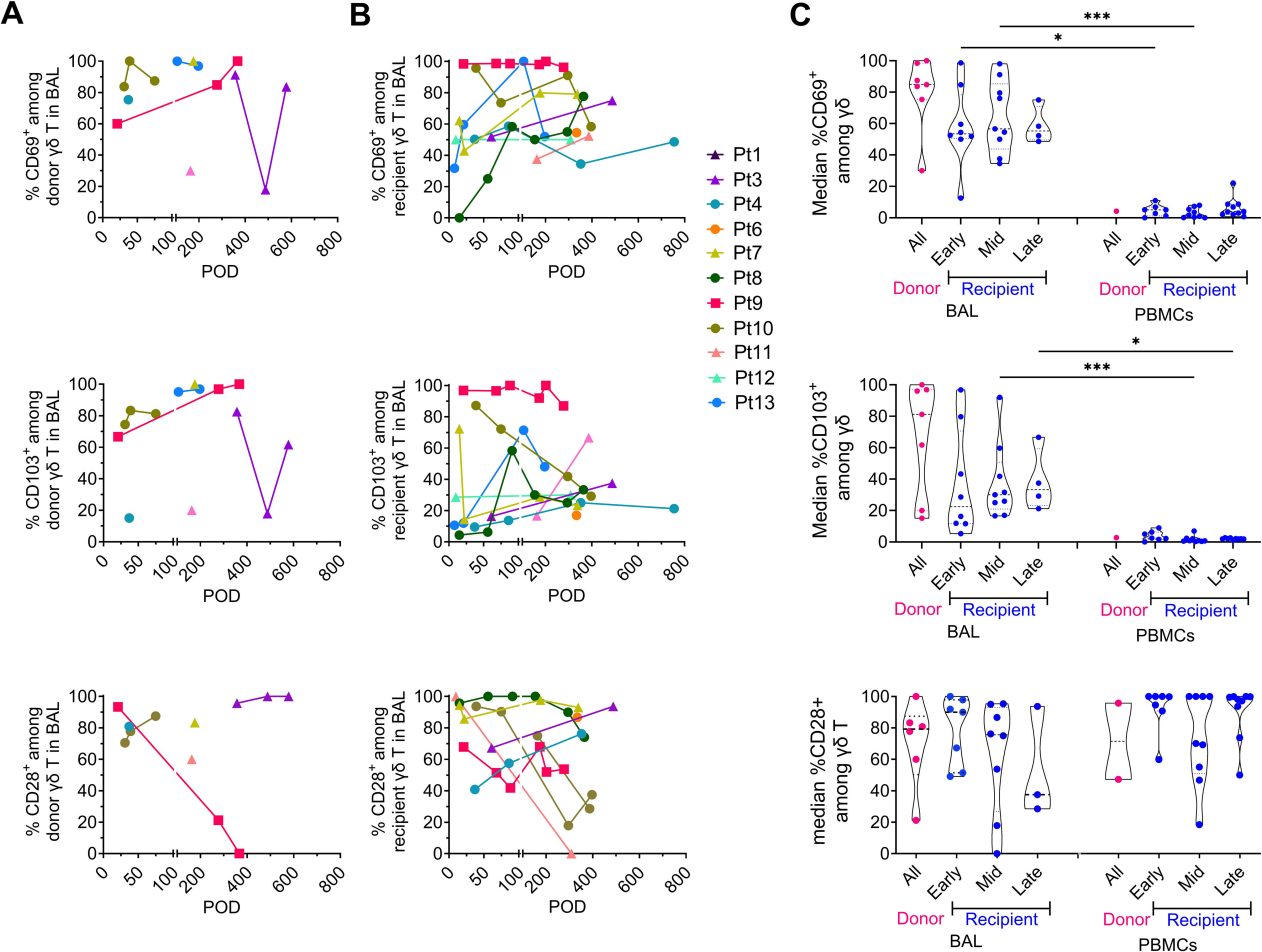
Tissue-residency and differentiation marker dynamics on donor- and recipient-derived γδ T cells in BAL and PBMCs. Longitudinal expression of CD69, CD103, and CD28 on **(A)** donor-derived and **(B)** recipient-derived γδ T cells in BAL. **(C)** Violin plots showing the median frequency of CD69+, CD103+, and CD28+ γδ T cells in BAL and PBMCs, stratified by cell origin (donor vs. recipient) and post-Tx interval (all intervals combined, early, mid, late). Kruskal–Wallis test with Dunn’s multiple comparisons was performed for panel **(C)** *p < 0.05, ***p <0.001.

We analyzed the tissue-resident memory (TRM) and effector T cell (Teff) phenotypes of donor and recipient γδ T cells in post-Tx BAL over time using canonical markers: CD103, CD69, and CD49a for TRM; CD28 and NKG2D for Teff (**Figure 4**; **Supplementary Figures 4**, **5**). As expected, TRM-associated markers were overall more abundant on γδ T cells in BAL than in peripheral blood for both donor and recipient cells (**Figures 4A–C**; **Supplementary Figures 5A–C**). In contrast, the abundance of CD28+ and NKG2D+ γδ T cells did not differ between BAL and blood (**Figure 4**, **Supplementary Figure 5**). Longitudinal analysis within the BAL compartment revealed a phenotypic shift among recipient γδ T cells: median levels of CD69+, CD103+, and CD49a+ cells increased from early to late post-Tx periods, while CD28+ cells decreased (**Figure 4C**, **Supplementary Figure 5C**). Unlike CD28, NKG2D expression on recipient γδ T cells in BAL tended to increase over time, suggesting a rise in cytotoxic potential concurrent with the acquisition of tissue residency. Together, these data identify a phenotypic transition from a Teff-like (CD28+) to a TRM-like (CD69+/CD103+/CD49a+) profile for recipient γδ T cells within the allograft. No associations were observed between donor or recipient TRM phenotypes and the incidence of rejection, *de novo* DSA development, or pulmonary function (data not shown).

### Rapid recipient γδ repopulation is associated with reduced CD103 expression, while circulating recipient TEM correlates with increased infection

**Figure 5:**
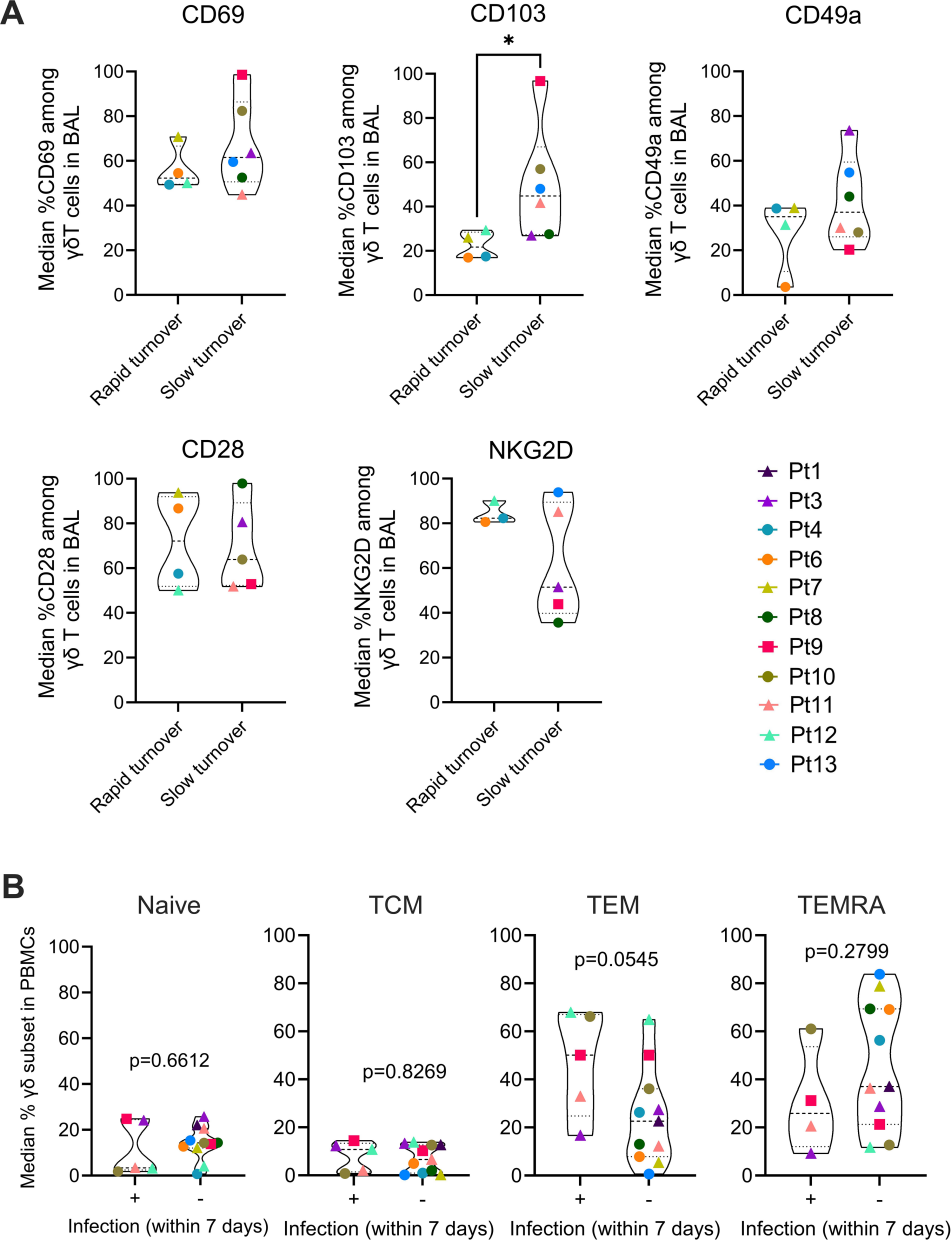
Association of recipient γδ T cell turnover with phenotype and infection. **(A)** Violin plots comparing the median frequency of CD69+, CD103+, CD49a+, CD28+, and NKG2D+ cells among recipient γδ T cells in BAL in patients with rapid vs. slow recipient γδ T cell turnover. **(B)** Distribution of recipient γδ T cell naïve and memory subsets in PBMCs—naïve, TCM, TEM, and TEMRA)—stratified by the presence (+) or absence (-) of a diagnosed infection within seven days of sampling. Unpaired two-tailed t test and Mann-Whitney test were performed for **(A, B)** *p < 0.05.

We next asked whether recipient γδ T cells that rapidly repopulate the allograft differ in TRM and Teff phenotypes. Comparison of the rapid- and slow-replacement groups (defined in **Figure 3**) revealed that recipient γδ T cells in the rapid-replacement group exhibited a significantly lower frequency of CD103+ cells, with similar non-significant trends for CD69 and CD49a (**Figure 5A**). This suggests that rapidly infiltrating recipient γδ T cells in BAL acquire a canonical TRM phenotype more slowly. In contrast, CD28 expression did not differ between groups, while NKG2D showed a non-significant trend toward higher expression in the rapid-replacement group. Using standard naïve and memory T cell markers (CD45RA vs CCR7; **Supplementary Figure 4**), we also analyzed the subset dynamics of donor and recipient γδ T cells in post-Tx PBMCs and BAL. We quantified the percentages of naïve, TCM, TEM and TEMRA cells. No individual subset was associated with the incidence of rejection, *de novo* DSA development, or pulmonary function. However, we observed a trend indicating that higher levels of circulating recipient γδ TEM cells​, but not naïve, TCM or TEMRA cells, were associated with infection onset (p=0.0545, **Figure 5B**). Because infection events did not always coincide with the PODs of sample collection, our analysis was restricted to infections that occurred within a seven-day window of a sampling time point. Together, these data suggest that circulating recipient γδ TEM cells may be primary mediators of blood-graft crosstalk, rapidly repopulating the lung allograft with a less TRM-like phenotype to participate in mucosal surveillance.

## Discussion

γδ T cells play complex and sometimes opposing roles in solid organ transplantation. Unlike αβ T cells, they lack intrinsic alloreactivity in standard mixed lymphocyte reactions (21). However, through functions ranging from immune surveillance and modulation to direct cytotoxicity (22), they can influence key post-Tx outcomes, including infection, malignancy, and rejection. In animal and human studies of kidney (23), skin (24), and liver (25, 26) transplantation, γδ T cells have been linked to graft acceptance, potentially via the production of IL-4 and IL-10. Conversely, in an orthotopic murine LuTx model, they cooperated with Th17 cells as primary producers of IL-17, promoting graft infiltration, rejection, and obliterative bronchiolitis (27). In LuTx patients, peripheral enrichment of NKG2C+ Vδ1+ γδ T cells has been associated with cytomegalovirus replication, suggesting antiviral activity (20). Despite the high load of donor lymphocytes within lung allografts and the resulting systemic and local chimerism, studies that clearly distinguish donor- from recipient-derived γδ T cells and define their distinct contributions to clinical outcomes in human LuTx are lacking. Our study addresses this gap by providing an unprecedented longitudinal resource that defines the abundance, chimerism, and phenotypic landscape of these cells and reveals their clinical significance.

We first established the baseline distribution of γδ T cells across compartments and transplant types (**Supplementary Figure 2**). While we observed no significant difference in γδ T cell abundance between pre-Tx donor and recipient BAL samples (**Supplementary Figure 2**), we found that bilateral LuTx recipients exhibited a significantly higher percentage of γδ T cells among total T cells in post-Tx BAL compared to single LuTx recipients. Several mechanisms may account for this finding. First, the increased allograft mass in bilateral LuTx introduces a larger mucosal surface area, potentially presenting a more sustained stress signal or a broader repertoire of allo-stimuli that favors the expansion or recruitment of γδ T cells in the mucosal niches. Second, bilateral procedures typically involve a greater cumulative duration of ischemic time and distinct kinetics of post-Tx mucosal injury and repair; given their role in maintaining epithelial integrity, γδ T cells may be preferentially recruited to these sites. Third, the total mucosal surface area in bilateral recipients may support a distinct microbial community compared to single-lung environments, where the presence of a native lung may fundamentally alter the competitive niche for γδ T cells. Notably, the difference in γδ T cell abundance between bilateral and single LuTx was not associated with divergent clinical outcomes, suggesting this observation reflects a baseline compartmental feature rather than a primary predictor of transplant success.

Because total γδ T-cell abundance does not distinguish donor from recipient origin, we next analyzed donor-recipient chimerism. In peripheral blood, higher donor graft-derived γδ T-cell chimerism tended to be associated with increased circulating donor γδ T-cell counts and a lower incidence of ACR. This direction of association is consistent with prior reports that donor graft-derived T cells can persist long-term in the lung allografts and correlate with less primary graft dysfunction (PGD) (4). These observations are reminiscent of our data after human intestinal transplantation (5), where the balance of αβ T cell-mediated GvH and HvG alloresponses within the allografts and hematopoietic system contribute to rejection outcomes, although these studies differ in organ system, compartment, and T cell populations analyzed, where γδ T cells lack intrinsic alloreactivity (21). In both contexts, it is possible that donor graft-derived γδ T cells migrating to peripheral blood, through cytotoxic effector functions, promoting lymphohematopoietic graft-versus-host responses (LGVHR) previously known to be mediated by αβ T cells (5). These responses may promote blood chimerism, counteract recipient HvG alloresponses, and thereby alleviate rejection. Thus, circulating donor γδ T cells may either contribute to, or serve as a biomarker of, more favorable graft outcomes.

While donor γδ T cell chimerism in peripheral blood may signify a favorable systemic immune environment, concurrent dynamics within the lung allograft are critical. We found that rapid recipient γδ T cell turnover in the BAL was associated with delayed infection and improved pulmonary function, but not with rejection. This pattern suggests that recipient γδ T cell repopulation reshapes mucosal immune surveillance in a compartment under constant microbial challenge, rather than serving as a direct marker of the alloreactive injury typically driven by αβ T cells, as observed in intestinal transplantation (5, 13). Notably, this clinical correlation aligns with the phenotypic data: patients with rapid recipient γδ turnover exhibited reduced expression of TRM markers, most prominently CD103. This suggests that the early wave of recipient γδ T cells repopulating the graft is more migratory and recruitment-prone, presumably comprised of circulating TEM counterparts, programmed for barrier surveillance and rapid effector function. This profile may underlie the observed enhancement in antimicrobial defense.

Regarding pulmonary function, our preliminary data (6) showed that impaired function, measured by %FEV1/baseline FEV1, significantly correlated with the enrichment of HvG-reactive recipient αβ T cells, but not microbial-reactive cells. Since a slower replacement of donor γδ T cells by recipient γδ T cells in BAL also significantly correlated with reduced pulmonary function, these observations raise the possibility that persistent donor γδ T cells in the lung allograft may act as APCs to prime HvG-reactive αβ T cell responses (28–30). Consistent with this speculation, donor γδ T cells remained detectable in BAL even at late timepoints, raising the question of how they persist. Both human intestines (12) and lungs (31) have been described as extramedullary sites that harbor hematopoietic stem and progenitor cells (HSPCs), which could replenish donor-derived T cells within the mucosal allograft via thymus-dependent or -independent mechanisms, a process requiring further investigation.

The seemingly contradictory findings that better graft outcomes are associated with both circulating donor γδ T cells and graft-repopulating recipient γδ T cells may reflect the multifunctional nature of γδ T cells and their compartmentalized functional priorities. Although γδ T cells lack intrinsic alloreactivity (21), our data suggest that circulating donor γδ T cells potentially modulate αβ T cell-mediated alloresponse by promoting LGVHR to counteract rejection; meanwhile, graft-repopulating recipient γδ T cells are likely prioritized for barrier surveillance against infection. A key unresolved question concerns the roles of graft-derived donor γδ T cells, which are presumably involved in distinct immune processes: a fraction of them may replenish the circulating pool to facilitate LGVHR, while others could act as APCs to prime HvG-reactive αβ T cells, thereby causing allograft dysfunction. Therefore, graft-derived donor γδ T cells may provide diverse contribution to graft outcomes that require further investigation utilizing multiomic profiling.

The cohort size and sampling density limit the statistical power for certain clinical endpoints, such as ACR and infection; therefore, all non-significant associations should be considered hypothesis-generating. Furthermore, our analysis did not resolve γδ T cell subsets (e.g., Vδ2+ vs. Vδ2-), antigen specificities, or functional states beyond surface phenotypes. This leaves open questions of which specific γδ T cell programs are the most relevant to mucosal defense versus alloimmune modulation. Future studies should employ γδ T cell receptor sequencing to define clonal dominance, persistence, and migration patterns, building on approaches that have proven informative for αβ T cells in transplantation (8). Functional assays combined with high-resolution phenotyping will be essential to link the observed γδ T cell dynamics to underlying mechanisms. Finally, in the current study, longitudinal inferences are limited by the small cohort size, non-uniform sampling intervals, and a limited number of paired immune and clinical timepoints, which constrained our ability to account for potential confounders such as infectious complications and changes in immunosuppression. Future research in an expanded patient cohort should incorporate multivariable analyses to account for donor and recipient demographics, immunosuppressive regimens, and long-term outcomes.

Together, our findings provide a longitudinal framework and resource for understanding the compartmentalized dynamics of donor- and recipient-derived γδ T cells after human lung transplantation and their association with infection, graft function, and rejection. Deciphering these γδ T cell dynamics may pave the way for novel biomarkers and therapeutic interventions aimed at improving long-term lung allograft outcomes.

## Data Availability

The raw data supporting the conclusions of this article will be made available by the authors, without undue reservation.

## Supplementary material

Supplementary Figure 1 Clinical outcomes after LuTx, stratified by transplant type. Kaplan-Meier analysis of **(A)** patient survival, **(B)** ACR-free survival, **(C)** *de novo* DSA-free survival, and **(D)** infection-free survival, comparing single and bilateral LuTx recipients. Kaplan–Meier analysis with log-rank (Mantel–Cox) test was performed for panels **(A–D)**.

Supplementary Figure 2 Presence of γδ T cells before LuTx. Frequency of donor (red)- and recipient (blue)-derived γδ T cells among CD3^+^ T cells in lung tissue, bronchoalveolar lavage (BAL), lung-draining lymph node (LLN), and peripheral blood mononuclear cells (PBMCs) pre-Tx when samples were available for research. Mann-Whitney test was performed between donor and recipient LLN subgroups with at least two samples available for each subgroup. ns: no significance.

Supplementary Figure 3 Association of recipient γδ T cell turnover in BAL with clinical outcomes and transplant characteristics. Kaplan-Meier analysis of **(A)** patient survival and **(B)** *de novo* DSA-free survival, stratified by recipient γδ T cell turnover in BAL (rapid vs. slow; median cutoff, 92%). Linear regression assessing the relationships between the median percentage of recipient γδ T cell repopulation in BAL and **(C)** recipient age at transplantation or **(D)** donor age. Kaplan–Meier analysis with log-rank (Mantel–Cox) test was performed for panels **(A)** and **(B)**. Simple linear regression with ANOVA F-test was performed for panels **(C)** and **(D)**.

Supplementary Figure 4 Representative gating of donor- and recipient-derived γδ T cell phenotypes in PBMCs and BAL after LuTx. Gating strategy for **(A)** PBMCs (Pt8 POD580) and **(B)** BAL (Pt4 POD37). CD3+γδTCR+ cells were first stratified into recipient- and donor-derived populations using HLA allotype-specific staining. Expression of CD69 vs. CD103, CD69 vs. CD49a, CD28 vs. NKG2D, and CD45RA vs. CCR7 was then assessed within recipient (top row) and donor (bottom row) γδ T cells. Healthy control PBMC sample was included as a reference of flow cytometric gating for each patient sample (data not shown).

Supplementary Figure 5 CD49a and NKG2D expression on donor- and recipient-derived γδ T cells in BAL and PBMCs. Longitudinal frequency of CD49a+ (top) and NKG2D+ (bottom) cells among **(A)** recipient-derived and **(B)** donor-derived γδ T cells in BAL, plotted against POD. **(C)** Violin plots showing the median frequency of CD49a+ (top) and NKG2D+ (bottom) γδ T cells in BAL and PBMCs, stratified by cell origin (donor vs. recipient) and post-Tx interval (all intervals combined, early, mid, late). Kruskal–Wallis test with Dunn’s multiple comparisons was performed for panel **(C)**. **p<0.01, ***p<0.001, ****p<0.0001.

Supplementary Table 1 Clinical characteristics and outcomes of the lung transplant recipient cohort. Summary table of recipient demographics, transplant indication, transplant type, rejection episodes, infectious complications, BOS onset and stage, CLAD onset and subtype, and death or cause of death for each patient included in the study. BOS and CLAD onset are reported by postoperative day (POD), with BOS stage and CLAD subtype indicated where applicable. Definitions for BOS (32) and CLAD (33) were adapted from prior literature.

Supplementary Table 2 HLA-A, B typing and anti-HLA allotype antibodies used to distinguish donor from recipient cells in lung transplant recipients. HLA-A09 is a broad antigen HLA-A serotype that recognized the HLA-A23 and HLA-A24 serotypes. HLA-A28 is a broad antigen HLA-A serotype that recognized the HLA-A68 and HLA-A69 serotypes. HLA-B12 is a broad antigen HLA-B serotype that recognized the HLA-B44 and HLA-B45 serotypes.

Supplementary Table 3 List of flow cytometric antibodies used in the study.
