# Supplementary material for: Longitudinal profiling of γδ T cell dynamics after human lung transplantation links donor and recipient subsets to clinical outcomes
Source: Front Immunol. 2026 Apr 16;17:1811464. doi: 10.3389/fimmu.2026.1811464 (PMC13128400; doi:10.3389/fimmu.2026.1811464)
Supplement: Supplementary Figure 1 — Clinical outcomes after LuTx, stratified by transplant type. Kaplan-Meier analysis of (A) patient survival, (B) ACR-free survival, (C) de novo DSA-free survival, and (D) infection-free survival, comparing single and bilateral LuTx recipients. Kaplan–Meier analysis with log-rank (Mantel–Cox) test was performed for panels (A–D). [file SupplementaryFile1.docx]

**Supplemental figures.**

**Fig. S1: Clinical outcomes after LuTx, stratified by transplant type.** Kaplan-Meier analysis of (A) patient survival, (B) ACR-free survival, (C) de novo DSA-free survival, and (D) infection-free survival, comparing single and bilateral LuTx recipients. Kaplan–Meier analysis with log-rank (Mantel–Cox) test was performed for panels A-D.


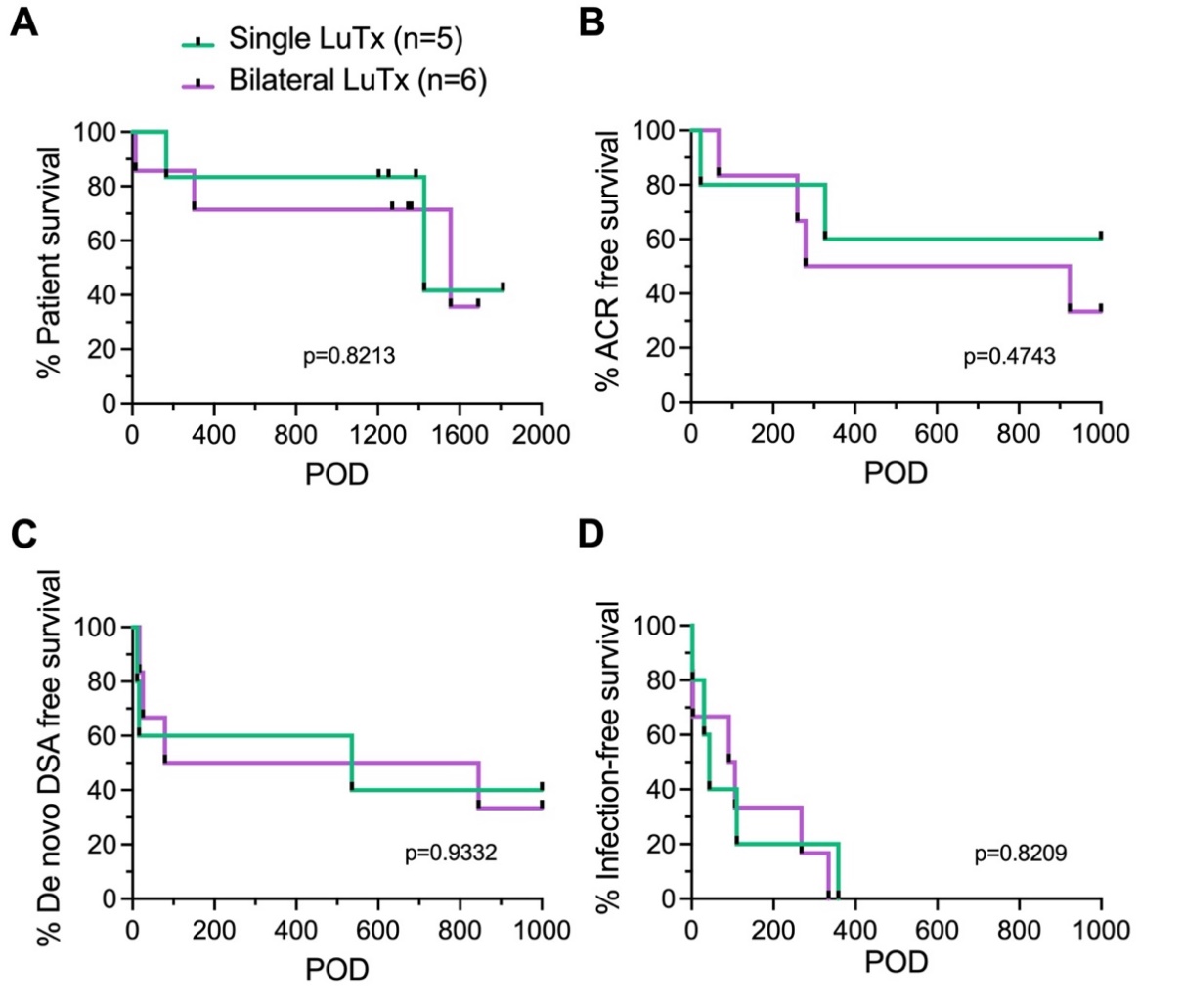


**Fig. S2: Presence of γδ T cells before LuTx.** Frequency of donor (red)- and recipient (blue)-derived γδ T cells among CD3^+^ T cells in lung tissue, bronchoalveolar lavage (BAL), lung-draining lymph node (LLN), and peripheral blood mononuclear cells (PBMCs) pre-Tx when samples were available for research. Mann-Whitney test was performed between donor and recipient LLN subgroups with at least two samples available for each subgroup. ns: no significance.

**
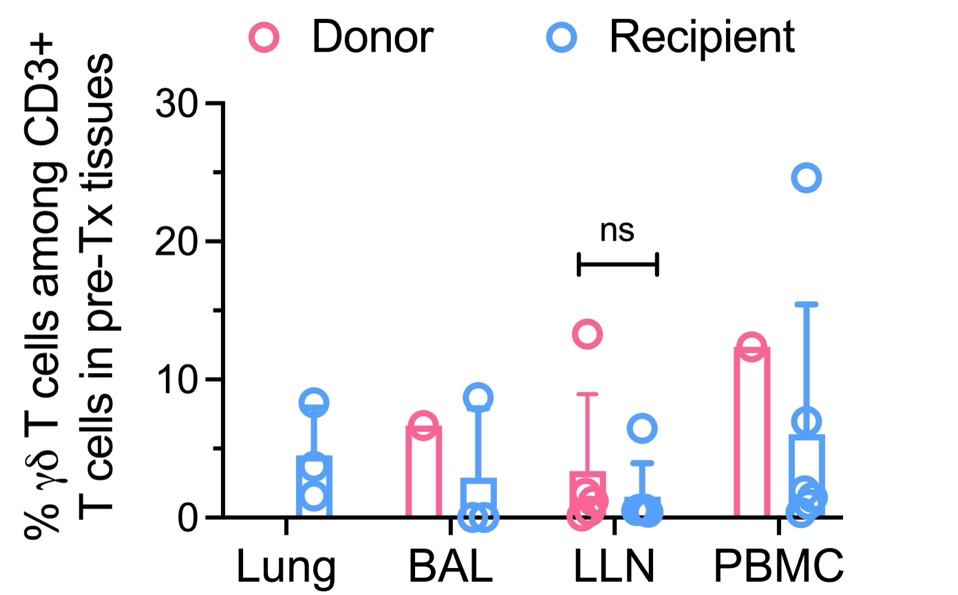
**

**Fig. S3: Association of recipient γδ T cell turnover in BAL with clinical outcomes and transplant characteristics.** Kaplan-Meier analysis of (A) patient survival and (B) de novo DSA-free survival, stratified by recipient γδ T cell turnover in BAL (rapid vs. slow; median cutoff, 92%). Linear regression assessing the relationships between the median percentage of recipient γδ T cell repopulation in BAL and (C) recipient age at transplantation or (D) donor age. Kaplan–Meier analysis with log-rank (Mantel–Cox) test was performed for panels A and B. Simple linear regression with ANOVA F-test was performed for panels C and D.


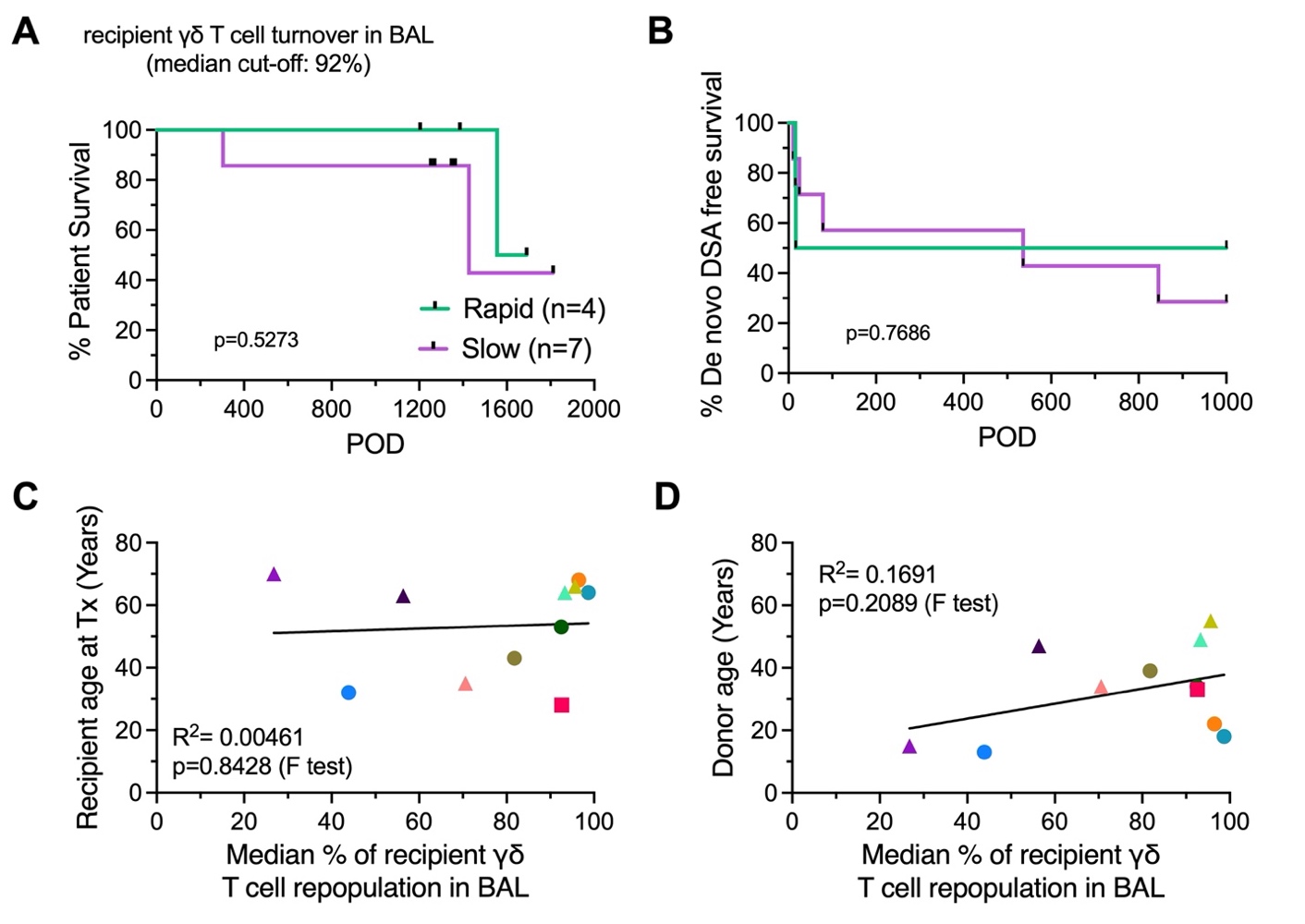


**Fig.S4: Representative gating of donor- and recipient-derived γδ T cell phenotypes in PBMCs and BAL after LuTx.** Gating strategy for (A) PBMCs (Pt8 POD580) and (B) BAL (Pt4 POD37). CD3+γδTCR+ cells were first stratified into recipient- and donor-derived populations using HLA allotype-specific staining. Expression of CD69 vs. CD103, CD69 vs. CD49a, CD28 vs. NKG2D, and CD45RA vs. CCR7 was then assessed within recipient (top row) and donor (bottom row) γδ T cells. Healthy control PBMC sample was included as a reference of flow cytometric gating for each patient sample (data not shown).


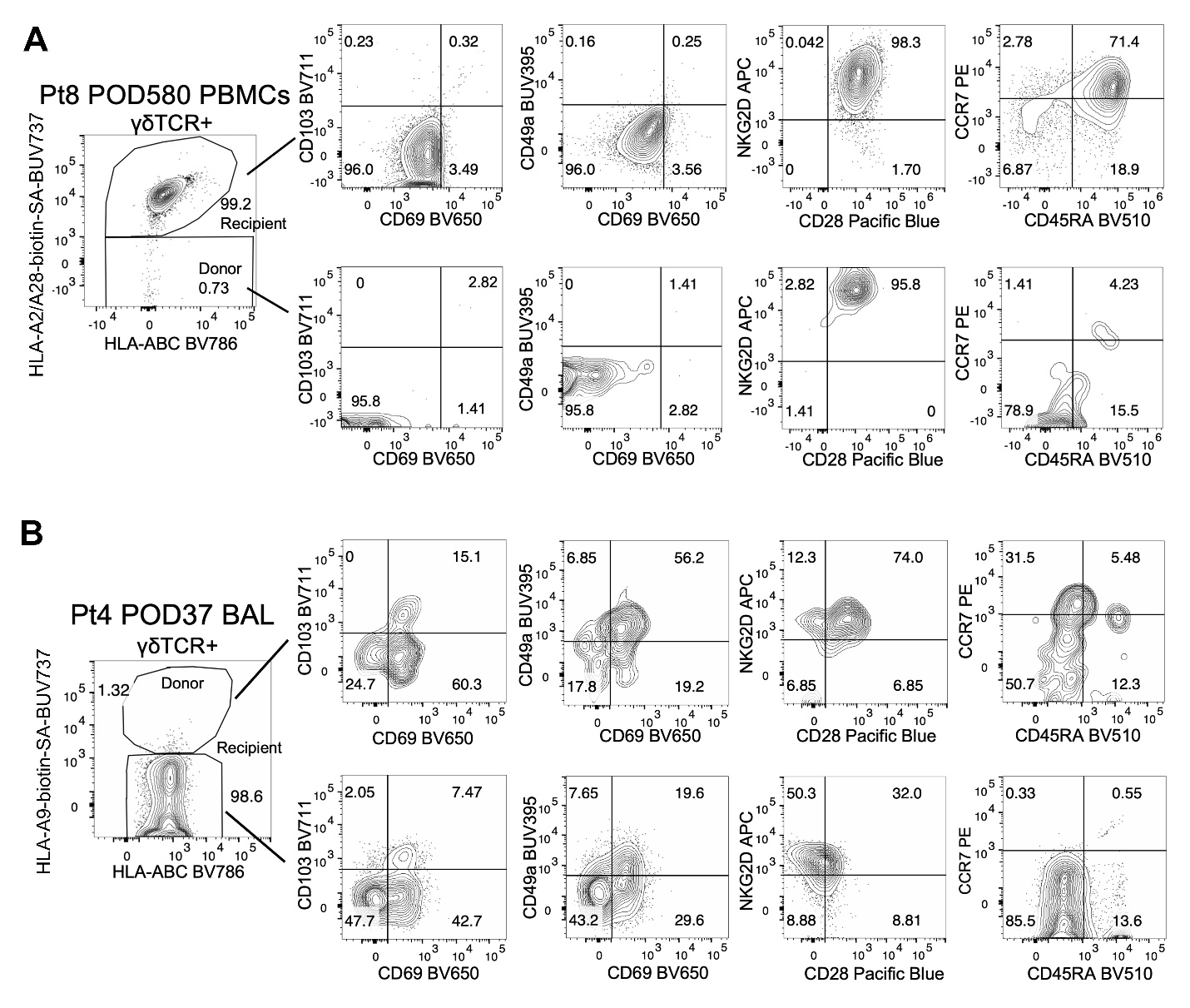


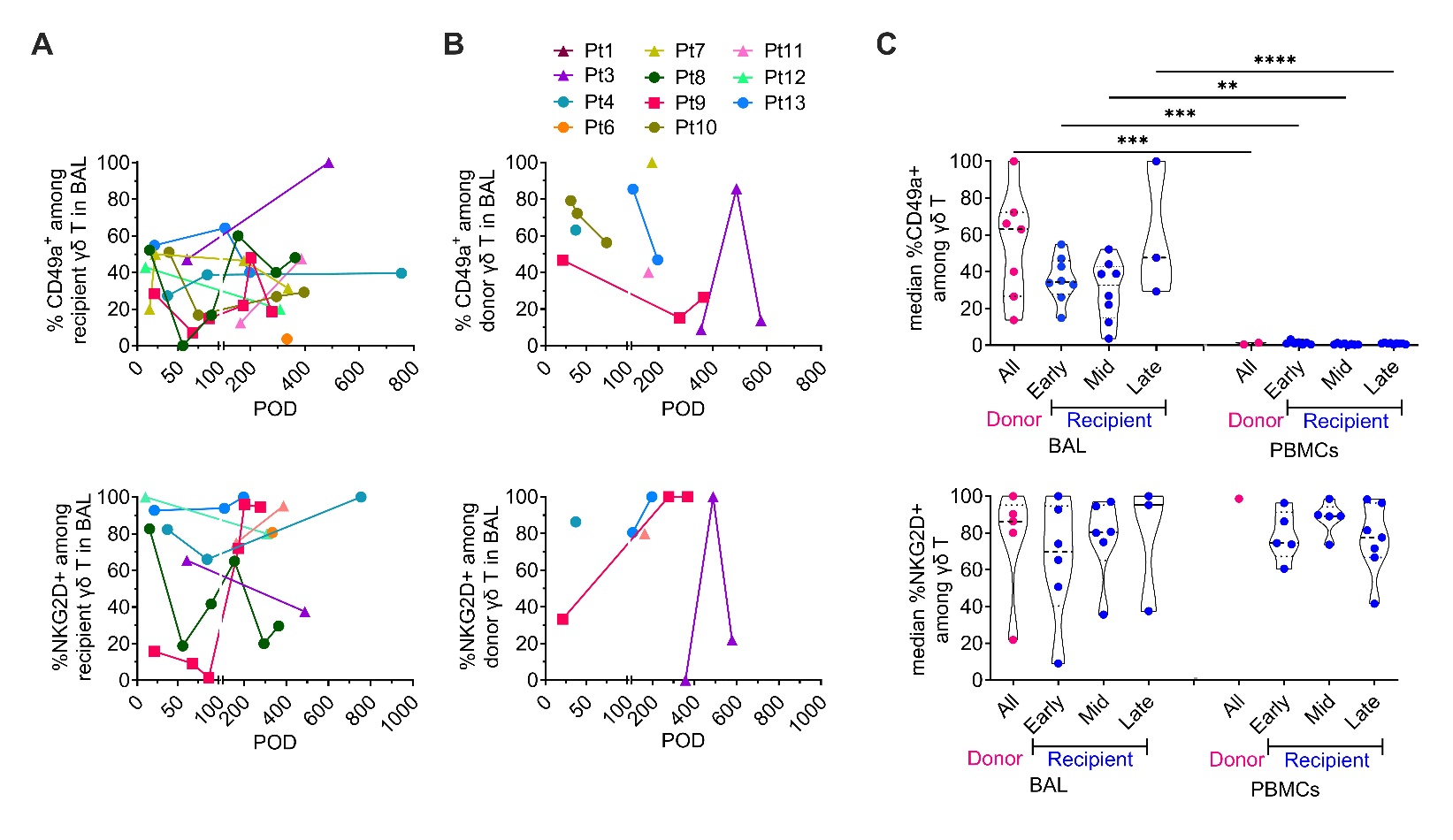
**Fig. S5: CD49a and NKG2D expression on donor- and recipient-derived γδ T cells in BAL and PBMCs.** Longitudinal frequency of CD49a+ (top) and NKG2D+ (bottom) cells among (A) recipient-derived and (B) donor-derived γδ T cells in BAL, plotted against POD. (C) Violin plots showing the median frequency of CD49a+ (top) and NKG2D+ (bottom) γδ T cells in BAL and PBMCs, stratified by cell origin (donor vs. recipient) and post-Tx interval (all intervals combined, early, mid, late). Kruskal–Wallis test with Dunn’s multiple comparisons was performed for panel C. **p<0.01, ***p<0.001, ****p<0.0001.

**Table S1:** **Clinical characteristics and outcomes of the lung transplant recipient cohort.** Summary table of recipient demographics, transplant indication, transplant type, rejection episodes, infectious complications, BOS onset and stage, CLAD onset and subtype, and death or cause of death for each patient included in the study. BOS and CLAD onset are reported by postoperative day (POD), with BOS stage and CLAD subtype indicated where applicable. Definitions for BOS (32) and CLAD (33) were adapted from prior literature.

| **Pt #** | **Recipient Age at Tx (yrs)** | **Recipient Sex** | **Indication(s) for Transplant** | **Type of LuTx** | **Rejection** | **Infection** | **BOS onset and stage** | **CLAD onset and subtype** | **Death/Cause of Death** |
| --- | --- | --- | --- | --- | --- | --- | --- | --- | --- |
| 1 | 63 | F | Pulmonary emphysema | Single | No | Kiebsiella, COVID19, CMV | POD1699:  Stage 2. POD1874: Stage 1. | No | No |
| 2 | 71 | M | Chronic atrial fibrillation | Single | No | No | No | No | POD166: cardiopulmonary arrest secondary to complications related to hypoxia and atrial fibrillation and rapid ventricular response |
| 3 | 70 | M | Interstitial lung disease | Single | POD327-  A0B1R | Stenotrophomonas maltophilia, Lomentospora | POD17/31/50/71/92/561/575: Stage 1. POD610/645/736/757/799/904/1024/1185/1333: Stage 2. POD989/1394: Stage 3. | POD 561: Stage 1 CLAD (BOS subtype); POD610: Stage 2 CLAD (BOS subtype) | POD1427: respiratory failure. Contributory causes: gastrointestinal hemorrhage and skin malignancy |
| 4 | 64 | F | Interstitial lung disease, Pulmonary hypertension | Bilateral | POD924-  minimal ACR (A1B0) | Aspergillus, Pseudomonas, Aeruginosa, Klebsiella, Mycobacterium avium, CMV, BKV | POD26/37/943/987/1041: Stage 1. | POD 943: Stage 1 CLAD (indetermined subtype) | POD1556: respiratory failure. Contributory cause: liver failure |
| 5 | 63 | F | Pulmonary hypertension | Bilateral | No | No | No | No | POD16-: bowel perforation with multisystem organ failure |
| 6 | 68 | M | Pulmonary hypertension | Bilateral | No | Stenotrophomonas maltophilia, CMV | No | No | No |
| 7 | 66 | M | Interstitial lung disease, Chronic obstructive pulmonary disease, Idiopathic pulmonary fibrosis, Bronchiolitis obliterans with organizing pneumonia | Single | POD23-  mild ACR (A2B0) | Aspergillus | POD112: Stage 2. POD126/966: Stage 1. | No | No |
| 8 | 53 | F | Idiopathic pulmonary fibrosis, Sjogren’s related nonspecific interstitial pneumonitis, Severe pulmonary hypertension | Bilateral | POD259-  Minimal ACR (A1B0) | CMV, pluralibacter | No | No | No |
| 9 | 28 | M | Cystic fibrosis | Bilateral+ Liver | POD4-  mild/moderate ACR in liver.  POD67-  mild ACR in lung  (A2B1R)  POD69-  mild ACR in liver  POD80-  minimal ACR in liver  POD172-  minimal ACR in lung (A1BX) | Scedosporium apiospermum, Enterobacter cloacae, Staph aureus, EBV | POD 32/38/45: Stage 2. POD 87/122/311: Stage 1. | No | No |
| 10 | 43 | F | Pulmonary hypertension | Bilateral | No | Candida tropicalis, Rhino, Enterovirus, CMV | POD 57/64/73: Stage 1. | No | No |
| 11 | 35 | M | Interstitial lung disease related to connective tissue disease, Systemic sclerosis | Single | No | Staph aureus, CMV, E. coli, Rhizopus spp, Coronavirus HKU1 | POD 20/27/43: Stage 2. POD 48/71/134: Stage 1. | No | No |
| 12 | 64 | F | Interstitial lung disease, Idiopathic pulmonary fibrosis | Single | No | HBV, HCV, Penicillium, Parainfluenza virus 1, Actinomyces | No | No | No |
| 13 | 32 | M | Pulmonary sarcoidosis | Bilateral | POD279-  mild ACR (A2BX) | CMV, EBV, VZV, Measles, Rubella, Candida, TB, Pseudomonas aeruginosa, Mycobacterium, Parainfluenza | POD 34/42: Stage 1. | No | POD303: multisystem organ failure |

**Table S2**: HLA-A, B typing and anti-HLA allotype antibodies used to distinguish donor from recipient cells in lung transplant recipients. HLA-A09 is a broad antigen HLA-A serotype that recognized the HLA-A23 and HLA-A24 serotypes. HLA-A28 is a broad antigen HLA-A serotype that recognized the HLA-A68 and HLA-A69 serotypes. HLA-B12 is a broad antigen HLA-B serotype that recognized the HLA-B44 and HLA-B45 serotypes.

| Pt# | Recipient HLA-A, B type | Donor HLA-A, B type | HLA allotype-specific antibodies used to distinguish between recipient and donor cells |
| --- | --- | --- | --- |
| Pt1 | A03 / A29 | A30 / A36 | Anti-HLA A3 APC |
|  | B27 / B44 | B42 / B53 |  |
| Pt2 | A03 / A29 | A30 / A36 | Anti-HLA A3 APC, Anti-HLA B12 FITC |
|  | B61 / B44 | B42 / B53 |  |
| Pt3 | A02 / A03 | A01 / A03 | Anti-HLA A2/A28 biotin, Anti-HLA B8 FITC |
|  | B39 / B44 | B08 / B35 |  |
| Pt4 | A03 / A31 | A24 / A68 | Anti-HLA A3 APC, Anti-HLA A2/A28 biotin |
|  | B35 / B58 | B71 / B35 |  |
| Pt5 | A02 / A03 | A02 / A29 | Anti-HLA A3 APC, Anti-HLA B12 FITC |
|  | B35 / B61 | B35 / B44 |  |
| Pt6 | A34 / A74 | A02 / A24 | Anti-HLA A2/A28 biotin |
|  | B72 / B53 | B35 / B61 |  |
| Pt7 | A02 / A32 | A02 / A03 | Anti-HLA B12 FITC, Anti-HLA A3 APC |
|  | B44 / B52 | B13 / B51 |  |
| Pt8 | A33 / A68 | A31 / A32 | Anti-HLA A2/A28 biotin |
|  | B07 / B53 | B35 / B51 |  |
| Pt9 | A02 / A24 | A30 / A74 | Anti-HLA A2 FITC, Anti-HLA A09 biotin |
|  | B35 / B45 | B72 / B42 |  |
| Pt10 | A01 / A25 | A01 / A31 | Anti-HLA B7 APC |
|  | B08 / B18 | B07 / B08 |  |
| Pt11 | A01 / A68 | A33 / A66 | Anti-HLA A2/A28 biotin |
|  | B35 / B61 | B65 / B53 |  |
| Pt12 | A02 / A30 | A02 / A11 | Anti-HLA B12 FITC |
|  | B71 / B53 | B35 / B44 |  |
| Pt13 | A02 / A24 | A02 / A33 | Anti-HLA A9 FITC, Anti-HLA Bw4 biotin |
|  | B60 / B57 | B39 / B65 |  |
|  | Bw4 / Bw6 | Bw6 / Bw6 |  |

**Table S3** List of flow cytometric antibodies used in the study.

| Antibody | Source | Identifier |
| --- | --- | --- |
| Mouse anti-Human CD3 (clone SP34-2) PerCP-Cy5.5 | BD Biosciences | Cat#552852; RRID: AB_394493 |
| Mouse anti-Human CD4 (clone OKT4) AF700 | Tonbo | Cat#80-0048; RRID: AB_2621976 |
| Mouse anti-Human CD8 (clone SK1) APC-Cy7 | BD Biosciences | Cat#561945; RRID: AB_396892 |
| Mouse anti-Human CD8 (clone SK1) Biotin | BioLegend | Cat#344720; RRID: AB_2828353 |
| Mouse anti-Human CD14 (clone M5E2) FITC | BioLegend | Cat#982502; RRID: AB_2616906 |
| Mouse anti-Human CD14 (clone M5E2) APC-Cy7 | BioLegend | Cat#301820; RRID: N/A |
| Mouse anti-Human CD19 (clone SJ25C1) BUV496 | BD Biosciences | Cat#612938; RRID: AB_2870221 |
| Mouse anti-Human CD28 (clone CD28.2) Pacific Blue | BioLegend | Cat#302928; RRID: AB_10641279 |
| Mouse anti-Human CD45 (clone HI30) PE-CF594 | BD Biosciences | Cat#562279; RRID: AB_11154577 |
| Mouse anti-Human CD45RA (clone HI100) Brilliant Violet 510 | BioLegend | Cat#304142; RRID: AB_2561947 |
| Mouse anti-Human CD49a (clone SR84) BUV395 | BD Biosciences | Cat#742363; RRID: AB_2740721 |
| Mouse anti-Human CD56 (clone HCD56) Brilliant Violet 605 | BioLegend | Cat#318334; RRID: AB_2561912 |
| Mouse anti-Human CD69 (clone FN50) Brilliant Violet 650 | BioLegend | Cat#310934; RRID: AB_256315 |
| Mouse anti-Human CD103 (clone Ber-ACT8) Brilliant Violet 711 | BioLegend | Cat#350222; RRID: AB_2629651 |
| Mouse anti-Human CD197 (CCR7) (clone G043H7) PE | BioLegend | Cat#353204; RRID: AB_10913813 |
| Mouse anti-Human CD314 (NKG2D) (clone 1D11) APC | BioLegend | Cat#320808; RRID: AB_492962 |
| Mouse anti-Human HLA-A2/A28 (clone N/A) Biotin | One Lambda | Cat#BIH0037; RRID: N/A |
| Mouse anti-Human HLA-A2/A28 (clone REA142) FITC | Miltenyi Biotech | Cat#130-099-601; RRID: AB_2652040 |
| Mouse anti-Human HLA-A2 (clone BB7.2) FITC | BioLegend | Cat#343304; RRID: AB_1659245 |
| Mouse anti-Human HLA-A3 (clone GAP.A3) APC | Thermo Fisher Scientific | Cat#17-5754-42; RRID: AB_2573220 |
| Mouse anti-Human HLA-A9 (clone N/A) Biotin | One Lambda | Cat#BIH0964; RRID: N/A |
| Mouse anti-Human HLA-A9 FITC (clone N/A) | One Lambda | Cat#FH0964; RRID: N/A |
| Mouse anti-Human HLA-A29 (clone N/A) Biotin | One Lambda | Cat#BIH0155; RRID: N/A |
| Mouse anti-Human HLA-ABC (clone G46-2.6) BV786 | BD Biosciences | Cat#740982; RRID: AB_2740606 |
| Mouse anti-Human HLA-B8 (clone N/A) FITC | One Lambda | Cat#FH0536A; RRID: N/A |
| Mouse anti-Human HLA-B12 (clone REA138) FITC | Miltenyi Biotech | Cat#130-099-860; RRID: AB_2652098 |
| Mouse anti-Human HLA-Bw4 (clone REA274) Biotin | Miltenyi Biotech | Cat#130-123-859; RRID: AB_2889687 |
| Mouse anti-Human HLA-Bw6 (clone REA143) APC | Miltenyi Biotech | Cat#130-099-845; RRID: AB_2652026 |
| Streptavidin Alexa Fluor 594 | Thermo Fisher Scientific | Cat#S32356; RRID: N/A |
| Streptavidin BUV737 | BD Biosciences | Cat#564293; RRID: AB_2869560 |
| Mouse anti-Human γδTCR (clone IMMU510) PE-Cy7 | Beckman Coulter | Cat#B10247; RRID: N/A |
